# Perilunate Dislocations and Perilunate Fracture-Dislocations of the Carpus: A Retrospective Study of 16 Cases

**DOI:** 10.7759/cureus.113705

**Published:** 2026-07-31

**Authors:** Mohamed-Anas Zeroual, Abdessalam Achkoun, Mohamed Nassiri, Mohamed Habbab, Rachid Chafik

**Affiliations:** 1 Orthopedics and Traumatology Department A, Ibn Tofail Hospital, Mohammed VI University Hospital Center, Marrakesh, MAR; 2 Orthopedics and Traumatology Department A, Ibn Tofail Hospital, Mohammed VI University Hospital Center, Marrakech, MAR

**Keywords:** carpus, fracture-dislocation, internal fixation, perilunate dislocation, retrospective study, wrist injury, wrist surgery

## Abstract

Introduction

Perilunate dislocations and perilunate fracture-dislocations of the carpus are serious wrist injuries that have significant functional and socio-occupational consequences. The objective of this study was to describe the epidemiological, clinical, radiological, and therapeutic characteristics of these injuries and to evaluate their outcomes.

Methods

We conducted a retrospective study over five years, including patients over the age of 15 with a pure perilunate dislocation or a perilunate fracture-dislocation confirmed by standard wrist X-rays. Demographic, clinical, radiological, therapeutic, and follow-up data were collected and analyzed.

Results

Sixteen patients were included, including three patients (18.8%) with pure perilunate dislocation and 13 patients (81.2%) with perilunate fracture-dislocation. The mean age was 29 years. A marked male predominance was observed, with 14 male patients (87.5%) and two female patients (12.5%). Road traffic accidents were the leading mechanism of injury, accounting for nine cases (56.3%). Diagnosis was established using standard anteroposterior and lateral wrist radiographs. The mean time to treatment was 12 hours. All patients (100%) underwent open reduction and internal fixation. After a mean follow-up of 24 months, 12 patients (75%) had returned to their previous occupation, two patients (12.5%) required occupational reassignment, and two patients (12.5%) were unable to resume their previous occupational activity. The mean wrist flexion-extension arc corresponded to 72.5% of the contralateral side, grip strength reached 76% of the healthy side, and the mean Cooney score was 65/100.

Conclusion

Early diagnosis and prompt surgical management remain essential for achieving satisfactory functional outcomes following a perilunate injury. The time interval between the injury and reduction appears to be a major prognostic factor. Studies with larger sample sizes and longer follow-up are needed to evaluate long-term functional and radiographic outcomes.

## Introduction

Perilunate dislocation is defined as a complete loss of contact between the capitolunate, scapholunate, and lunotriquetral articular surfaces. It is a relatively rare injury, accounting for approximately 7% of traumatic wrist injuries [1,2]. These injuries are frequently missed during the acute phase despite significant anatomical displacement (reported in up to 25% of cases). They usually result from high-energy trauma and are often associated with severe osseous, articular, capsular, and ligamentous injuries, which may compromise functional outcomes [3,4].

Several classification systems have been proposed to characterize perilunate injuries. Among them, the Herzberg classification is widely used to categorize perilunate dislocations and fracture-dislocations according to the pattern of carpal instability and displacement [5].

The aim of this retrospective case series was to evaluate the epidemiological characteristics, clinical presentation, surgical management, and functional and radiographic outcomes of 16 patients with perilunate dislocations and perilunate fracture-dislocations of the carpus treated by open reduction and internal fixation at the Orthopedics and Traumatology Department A of Ibn Tofail Hospital, Marrakech, Morocco.

## Materials and methods

Study overview

This was a single-center retrospective study conducted at the Orthopedics and Traumatology Department A of Ibn Tofail Hospital in Marrakech. The study included patients hospitalized for perilunate dislocations or perilunate fracture-dislocations of the carpus who underwent surgical treatment over five years from March 2017 to March 2022.

Data collection and study population

All patients admitted to the Orthopedics and Traumatology Department A of Ibn Tofail Hospital for wrist trauma between March 2017 and March 2022 were screened for eligibility. Among the 170 patients admitted with wrist trauma during the study period, 16 were diagnosed with perilunate dislocations or perilunate fracture-dislocations of the carpus. Patients aged 15 years or older with complete medical records who underwent surgical treatment were included in the study. No eligible patients were excluded, as all identified cases had complete medical records and adequate follow-up. Relevant clinical records were retrospectively reviewed, and demographic, clinical, radiological, surgical, and functional outcome data were collected using a standardized data collection form (Appendix 1).

Radiological assessment

Radiographic evaluation was based on standard anteroposterior and lateral wrist radiographs. Additional Schneck I and II views were obtained when a scaphoid fracture was suspected. Perilunate injuries were classified according to the Herzberg classification [5].

Functional outcome assessment

Functional outcomes were assessed at the final follow-up using the modified Mayo Wrist Score (Cooney score) [1], the Patient-Rated Wrist Evaluation (PRWE) [6], and the Quick Disabilities of the Arm, Shoulder and Hand (QuickDASH) questionnaire [7]. The Cooney score evaluates wrist function based on pain, functional status, range of motion, and grip strength. Raw PRWE scores (range, 0-150) and raw QuickDASH scores (range, 11-55) were recorded for all patients; higher scores indicate greater pain and disability. All instruments were used in accordance with their published guidelines for questionnaire administration.

Statistical analysis

Statistical analysis was performed using IBM SPSS Statistics for Windows, Version 25.0 (IBM Corp., Armonk, NY, USA) and Microsoft Excel (Microsoft Corporation, Redmond, WA, USA). Categorical variables were summarized as frequencies and percentages. Continuous variables were expressed as means and ranges. Given the descriptive nature of the study and the limited sample size, no inferential statistical analyses were performed.

Ethics statement

In accordance with institutional policy, formal ethics committee approval was not required for this retrospective review of anonymized medical records. Patient confidentiality was maintained throughout the study by anonymizing all collected data. All clinical and radiographic images included in this article were fully anonymized and do not contain any patient-identifiable information.

## Results

Epidemiological characteristics

**Figure 1:**
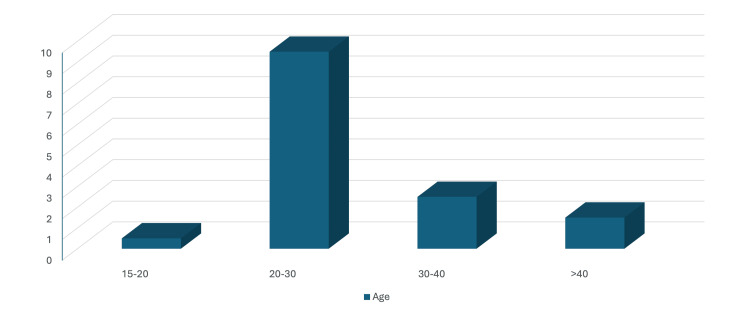
Age distribution of patients included in the study

A total of 170 patients with wrist trauma were admitted during the study period from March 2017 to March 2022, corresponding to an average of 34 cases per year. Perilunate injuries accounted for 9.4% of wrist trauma cases, with 16 patients identified. Perilunate dislocations and perilunate fracture-dislocations are injuries that typically occur in young adults. In our series, the mean age was 29.1 years (range: 19-55 years) (Figure 1).

A marked male predominance was observed, with 14 male patients (87%) and 2 female patients (13%). Among the 16 patients, 10 (62.5%) were manual workers, 4 (25.0%) were non-manual workers, and 2 (12.5%) were unemployed at the time of injury. Four mechanisms of injury were identified. Road traffic accidents were the most common cause, accounting for nine patients (56.3%), most frequently involving a dashboard impact. Other mechanisms included falls onto an outstretched hand in hyperextension in three patients (18.8%), occupational accidents in two patients (12.5%), and sports-related injuries in two patients (12.5%).

All patients were right-handed. The left wrist was affected in nine cases (56%), the right wrist in six cases (38%), and both wrists in one case (6%). The dominant hand was involved in seven patients (43.7%), whereas the non-dominant hand was affected in nine patients (56.2%).

Clinical characteristics

**Figure 2:**
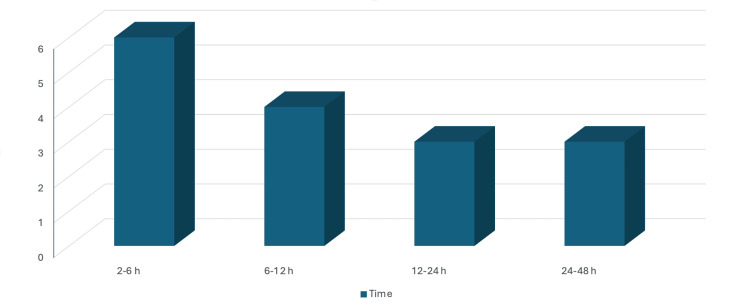
Distribution of patients according to time from injury to hospital admission

The time from injury to hospital admission ranged from two to 48 hours, with most patients presenting within two to six hours after trauma (Figure 2).

**Figure 3:**
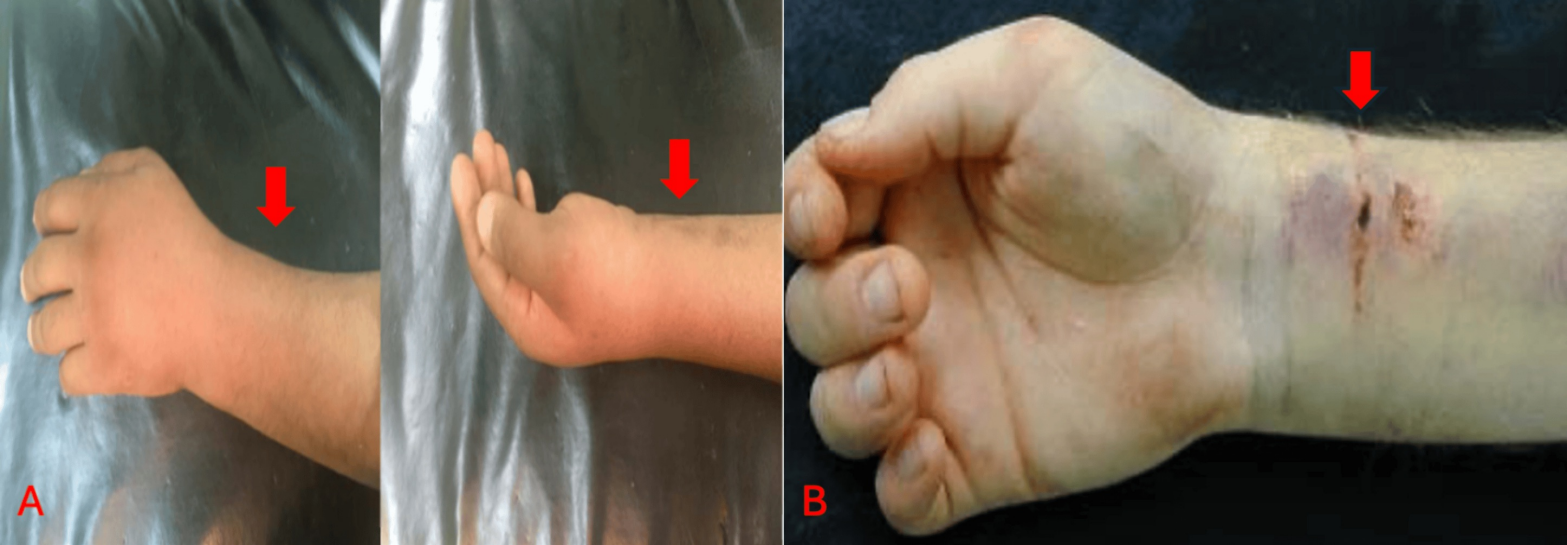
Clinical appearance of a patient presenting with a perilunate fracture-dislocation of the wrist (A) Marked soft-tissue swelling of the wrist (red arrows). (B) Wrist ecchymosis associated with visible deformity (red arrow).

The most common acute clinical findings were wrist pain, swelling, ecchymosis, deformity, and functional impairment (Figure 3A, 3B).

Regarding associated injuries, two patients (12.5%) presented with Gustilo-Anderson type I open injuries. Acute carpal tunnel syndrome associated with impaired thumb opposition and suggestive of median nerve compression was observed in two patients (12.5%). Associated fractures included one femoral fracture (6.3%), one acetabular fracture (6.3%), one forearm fracture (6.3%), and one mandibular fracture (6.3%). Associated head trauma was identified in two patients (12.5%).

Radiological evaluation

Diagnosis was based exclusively on standard anteroposterior and lateral wrist radiographs. Additional Schneck I and II views were obtained in cases of suspected scaphoid fracture. No computed tomography scans were performed in our series.

All injuries were classified according to the Herzberg classification (Table 1). Dorsal displacement was observed in 15 patients (93.8%), whereas volar displacement was identified in one patient (6.2%). Trans-scaphoid perilunate fracture-dislocation was the most common injury pattern, accounting for 10 cases (62.5%), including nine dorsal and one volar lesions. Pure perilunate dislocation was observed in three patients (18.8%), and perilunate dislocation associated with a radial styloid fracture was identified in three patients (18.8%). According to the Herzberg classification, 12 injuries (75%) were classified as Stage I and four (25%) as Stage II.

**Table 1:** Distribution of perilunate injuries according to direction of displacement and Herzberg stage Perilunate injuries were classified according to the Herzberg classification [4].

Direction of Displacement	Injury Pattern	Stage I	Stage II	Total
Dorsal	Pure perilunate dislocation	2	1	3
	Trans-scaphoid perilunate fracture-dislocation	7	2	9
	Perilunate dislocation with radial styloid fracture	2	1	3
Volar	Trans-scaphoid perilunate fracture-dislocation	1	0	1
Total		12	4	16

**Figure 4:**
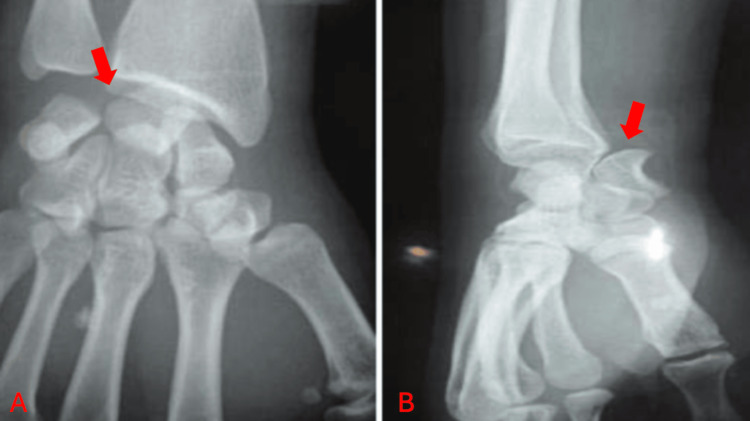
Anteroposterior and lateral wrist radiographs showing a pure perilunate dislocation classified as Herzberg stage IIb with dorsal displacement of the capitate (A) Anteroposterior view showing disruption of the normal carpal alignment with lunate dislocation (red arrow). (B) Lateral view demonstrating dorsal displacement of the capitate relative to the lunate (red arrow).

**Figure 5:**
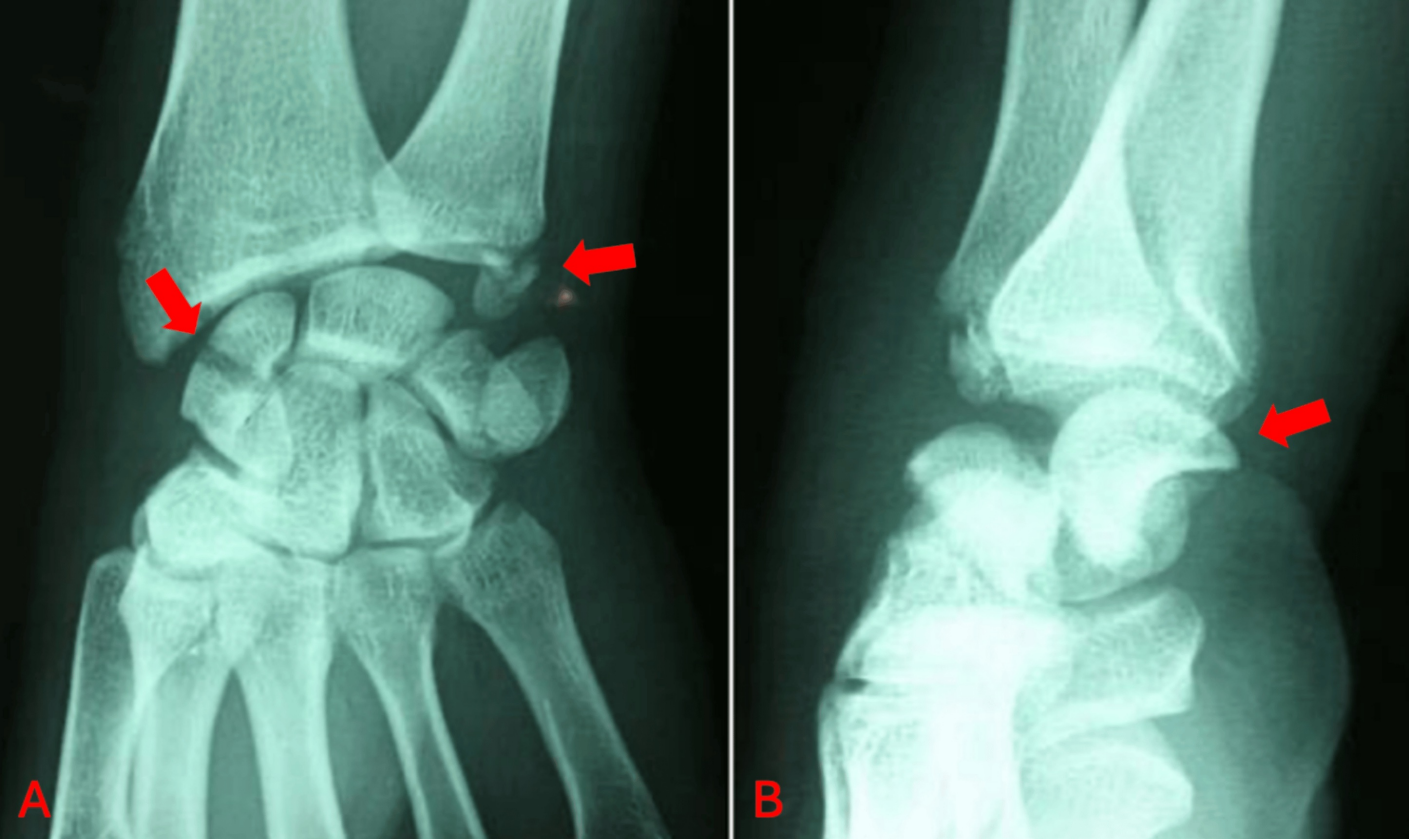
Anteroposterior and lateral wrist radiographs showing a transscaphoid perilunate fracture-dislocation associated with an ulnar styloid avulsion fracture (A) Anteroposterior view demonstrating the transscaphoid fracture and associated fracture of the ulnar styloid process (red arrows). (B) Lateral view showing dorsal displacement of the capitate relative to the lunate (red arrow).

Figures 4, 5 demonstrate representative radiographic findings, including pure perilunate dislocations and transscaphoid perilunate fracture-dislocations.

Surgical management

**Figure 6:**
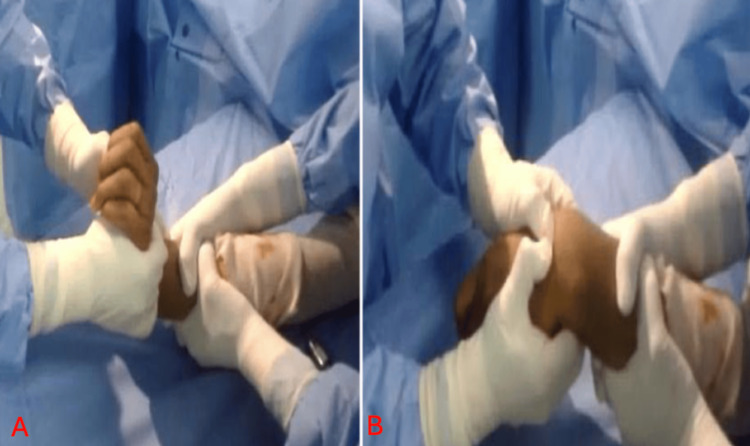
Closed reduction maneuver for perilunate dislocation using the Cooney–Böhler technique (A) Progressive axial traction of the wrist with hyperextension reproducing the injury mechanism. (B) Wrist flexion with maintained traction to reduce the capitate beneath the lunate.

All patients underwent attempted closed reduction in the operating room under anesthesia using the external manipulation technique described by Cooney and Böhler. This technique consists of progressive axial traction followed by wrist hyperextension, reproducing the mechanism of injury, and subsequent flexion with continued traction to reduce the capitate beneath the lunate (Figure 6).

Successful closed reduction was achieved in four patients (25%). However, all patients subsequently underwent open reduction and internal fixation. Closed reduction was attempted immediately after hospital admission under anesthesia in all patients. Excluding one patient with associated polytrauma who required delayed definitive surgical treatment after a seven-day stay in the intensive care unit, the mean time from injury to definitive surgical treatment was 12 hours (range: six to 48 hours). The surgical procedure was performed with the patient in the supine position under tourniquet control. General anesthesia was used in 10 patients (62.5%), whereas six patients (37.5%) underwent regional anesthesia. A dorsal approach was used in 12 patients (75%), an anterior approach in one patient (6.3%), and a combined approach in three patients (18.8%).

**Figure 7:**
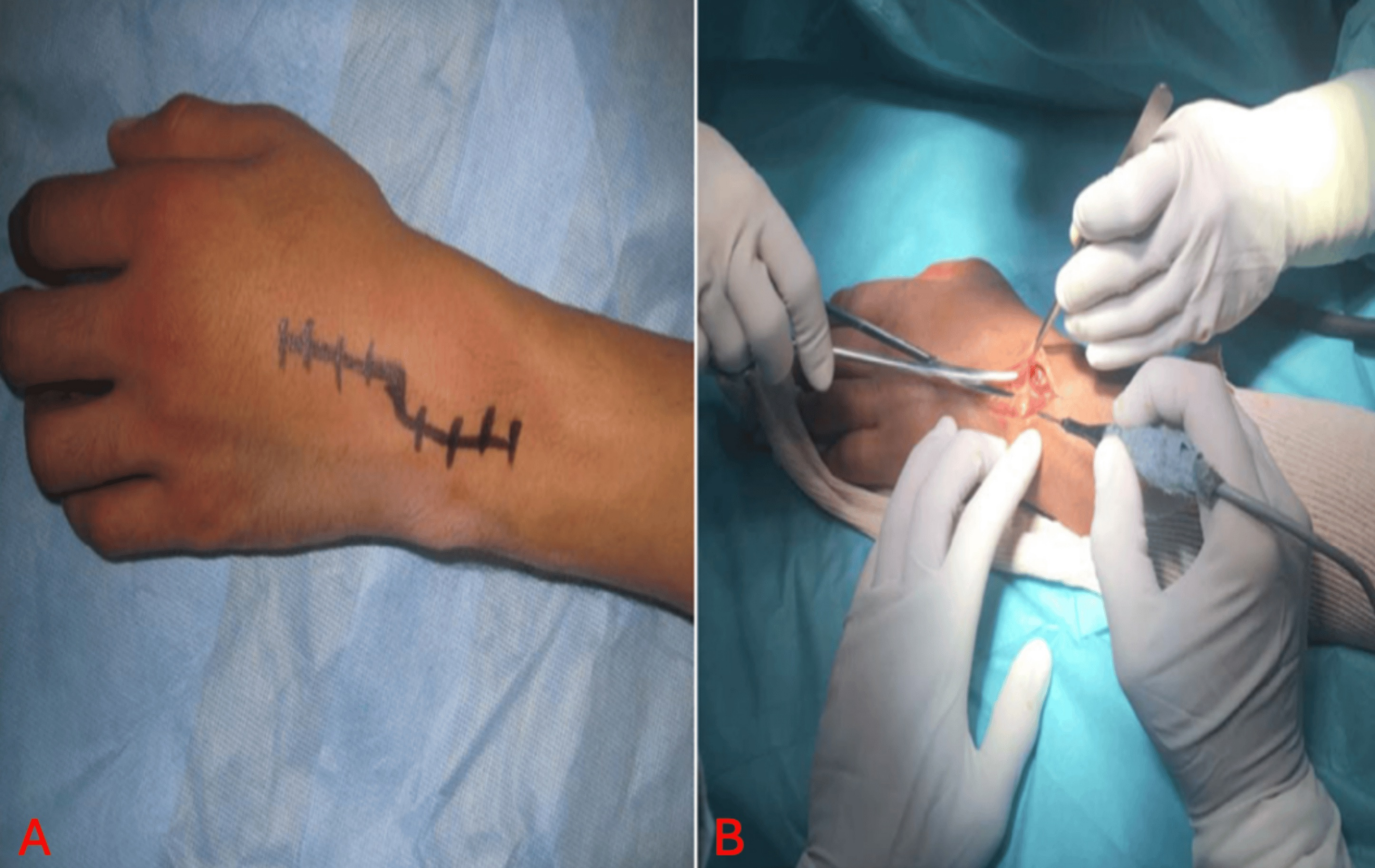
Dorsal surgical approach for open reduction and internal fixation of perilunate injuries (A) Dorsal surgical approach used for exposure of the carpus. (B) Five-centimeter posterior midline skin incision for the dorsal surgical approach.

**Figure 8:**
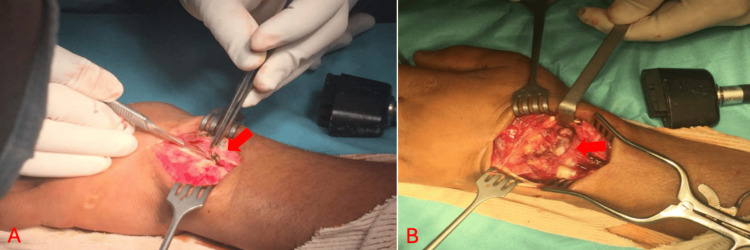
Opening of the extensor retinaculum during the dorsal surgical approach (A) Incision of the extensor retinaculum (red arrow). (B) Surgical exposure of the dorsal wrist structures following retinacular opening (red arrow).

**Figure 9:**
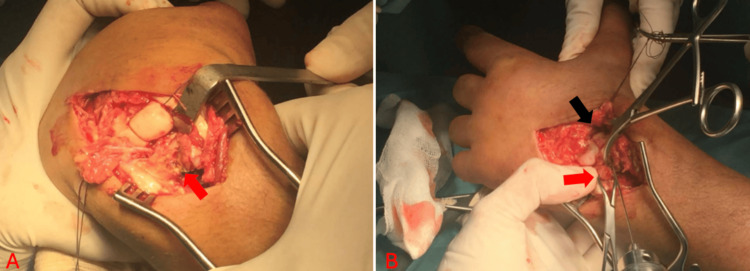
Intraoperative view of the carpus during surgical management of a perilunate injury (A) Exposed lunate identified during surgical exploration (red arrow). (B) Stabilization of the scaphoid fracture with a bone clamp (black arrow) and maintenance of the reduced lunate position with a second bone clamp (red arrow).

**Figure 10:**
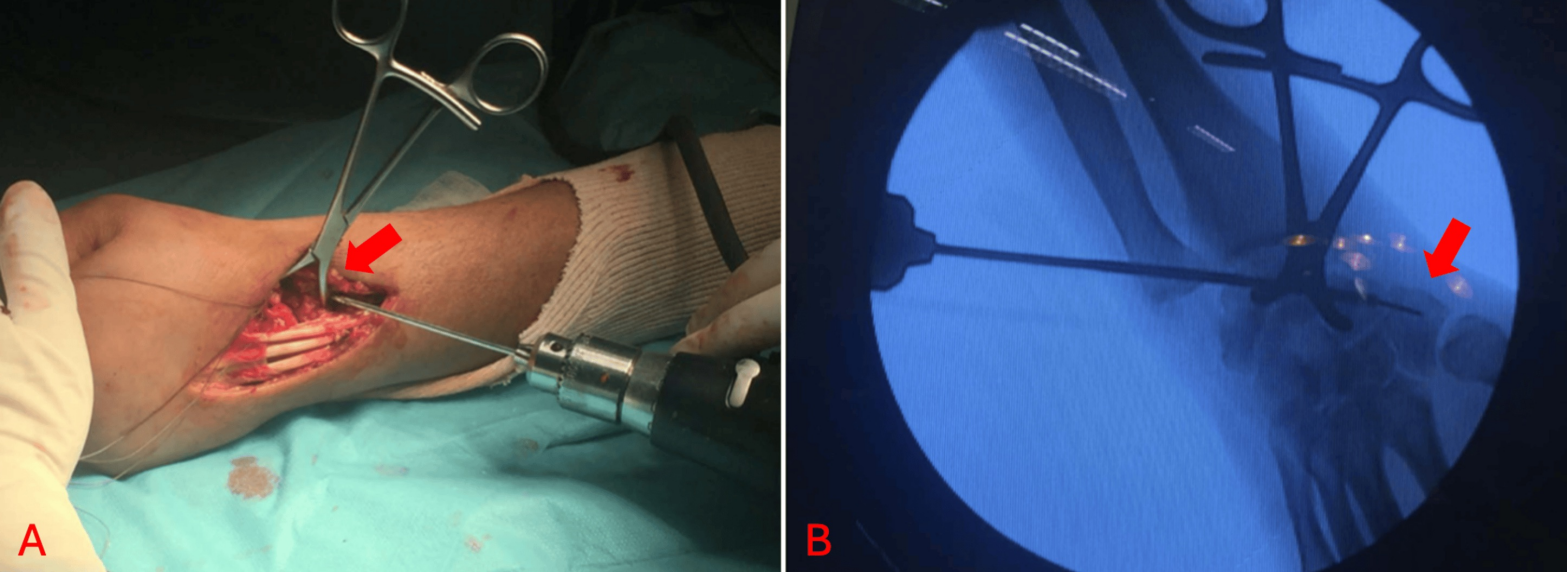
Preparation of the Herbert screw trajectory for scaphoid fracture fixation (A) Guide drilling of the scaphoid during screw trajectory preparation (red arrow). (B) Intraoperative fluoroscopic assessment confirming correct positioning of the drill within the scaphoid (red arrow).

Various fixation procedures were performed according to the injury pattern, and several patients required more than one fixation method. Scaphoid screw fixation using a Herbert screw was performed in four patients (25%) (Figures 7-10), scaphoid pinning in eight patients (50%), scapholunate pinning in five patients (31.3%), lunotriquetral pinning in 15 patients (93.8%), capitolunate pinning in three patients (18.8%), and radiolunate pinning in one patient (6.3%).

One patient (6.3%) underwent proximal row carpectomy.

Postoperative management and rehabilitation

All patients underwent immobilization with a plaster cast for six weeks, with an uncomplicated postoperative course. The mean time to removal of scaphoid pins was three months (range: two to six months). Rehabilitation was initiated after hardware removal, at five months for patients with scaphoid fractures and at four months for patients with other injury patterns. The rehabilitation protocol consisted of selective mobilization of the radiocarpal, midcarpal, and lateral carpal joints while avoiding intracarpal compression. The mean duration of rehabilitation was two months (range: 1.5 to four months), depending on patient compliance.

Functional and radiographic outcomes

The mean follow-up duration was 24 months (range: nine to 48 months). Among the 16 patients, 14 (87.5%) were employed at the time of injury. At the final follow-up, 12 patients (75%) had returned to their previous employment, including two patients (12.5%) after occupational reassignment. Two patients (12.5%) were unable to return to their previous occupation. All employed patients received three months of sick leave.

**Figure 11:**
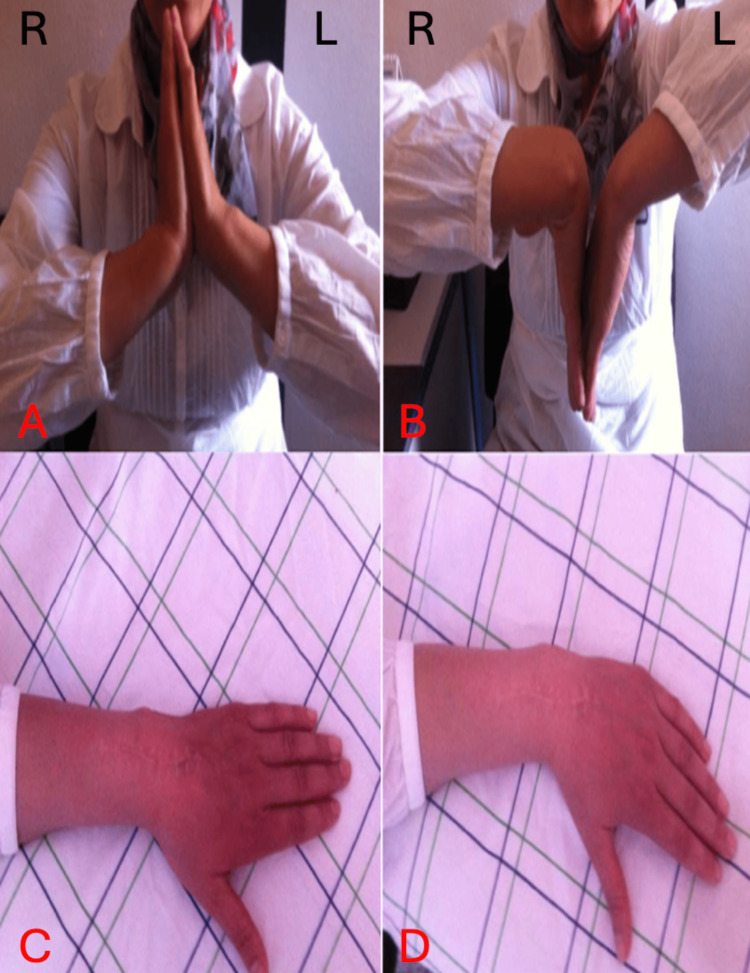
Comparative wrist range of motion in a representative patient with a left-sided perilunate injury (A) Wrist extension (dorsal flexion). (B) Wrist flexion (palmar flexion). (C) Ulnar deviation. (D) Radial deviation.

The mean wrist flexion-extension arc was 98°, corresponding to 72.5% of the contralateral wrist value (135°). The mean radioulnar deviation arc was 42°, representing 70% of the contralateral side (60°). Mean pronation-supination was 155°, corresponding to 86% of the unaffected side (180°). Mean grip strength measured by dynamometry was 38 kg compared with 50 kg on the healthy side, corresponding to 76%. Representative postoperative wrist mobility compared with the contralateral healthy side is illustrated in Figure 11.

Residual pain was rated at a mean of 3.5/10 on the visual analog scale. Functional assessment showed: mean Cooney score: 65/100; mean raw PRWE score: 40/150; mean raw QuickDASH score: 23/55.

Radiographic evaluation revealed one case (6.3%) of scapholunate diastasis and two cases (12.5%) with a scapholunate angle greater than 70°, consistent with dorsal intercalated segment instability. The carpal height ratio was reduced to less than 0.5 in two patients (12.5%). No cases of ulnar translation of the carpus were observed. Bone density changes were noted in seven patients (43.8%). Scaphoid nonunion was identified in two patients (12.5%). No cases of osteonecrosis were observed. Degenerative changes were identified in four patients (25%), including three patients (18.8%) with scapholunate advanced collapse wrist and one patient (6.3%) with scaphoid nonunion advanced collapse wrist.

## Discussion

In our series, the mean patient age was 29 years, which is consistent with previous reports in the literature. Lacour et al. [8], in a series of 60 cases, reported a mean age of 28 years, whereas Martinage et al. [9] reported a mean age of 35 years in a series of 14 patients. This young patient population can be explained by the mechanisms involved, which are typically high-energy trauma such as road traffic accidents, sports-related injuries, and falls from height. At this age, the distal radius demonstrates greater resistance compared with the carpal structures. During a high-energy trauma, a substantial amount of kinetic energy is transmitted to and absorbed by the carpus, resulting in severe capsuloligamentous and osteoarticular injuries. In contrast, in elderly patients, the distal radius is more susceptible to fracture, while in children, the injury pattern often involves physeal separation or distal radius fractures because the ligaments are stronger than the immature bone.

Male predominance is consistently reported across published series. Young active males are more frequently exposed to high-energy trauma compared with females, explaining the demographic distribution observed in perilunate injuries [10,11].

In our study, road traffic accidents were the most common mechanism of injury (nine cases), followed by falls from height and sports-related accidents. These findings are consistent with those reported by Martinage et al. [9] and Laporte et al. [19]. Although the right side is theoretically more frequently involved during road traffic accidents due to instinctive protective positioning, our series showed a predominance of left-sided injuries, with nine cases affecting the left wrist compared with six cases involving the right wrist.

Delayed diagnosis has been reported in 20-30% of cases in the literature [12], which was also observed in our study, with five delayed diagnoses. Clinical evaluation of an acute wrist injury should begin with careful assessment of the trauma mechanism, circumstances, and injury pattern. In chronic cases, attention should be directed toward loss of strength, reduced mobility, and persistent functional impairment.

On clinical examination, findings may include swelling, ecchymosis, and a characteristic fork-shaped deformity. During the acute phase, palpation usually reveals nonspecific wrist pain. In delayed presentations, pain tends to become more localized, particularly over the scapholunate interval, lunotriquetral interval, distal radioulnar joint, or scaphoid region. Associated vascular complications are rare.

The diagnosis of perilunate dislocations and perilunate fracture-dislocations is primarily radiographic. Clinical deformity of the wrist is nonspecific and does not allow definitive diagnosis. The difficulty of radiographic interpretation contributes to missed or delayed diagnoses. In our series, all patients underwent standard anteroposterior and lateral wrist radiographs, with additional scaphoid views (Schneck I or II) obtained in cases of suspected trans-scaphoid injuries. Trans-scaphoid patterns represent approximately 50% of cases reported in the literature. Volar perilunate dislocation is considerably less frequent, accounting for only 3% of cases in the Herzberg series.

Several authors have reported that early management is associated with improved functional outcomes [5,8]. Multiple studies have demonstrated that closed reduction alone has a high failure rate and cannot be considered a definitive treatment strategy, supporting surgical management as the recommended approach [2]. In our series, emergency closed reduction using external manipulation was successful in only 25% of cases, consistent with previous reports. Adkinson et al. [13] demonstrated that only 40% of patients maintained the initial reduction after immobilization in a plaster cast. In our series, no patient was treated exclusively with conservative management.

The objectives of surgical treatment are threefold: restoration of reduction, achievement of carpal stability, and management of associated fractures. However, the optimal surgical strategy remains controversial. The choice of surgical approach depends on several factors, including signs of median nerve compression, which may require a volar approach, and associated scaphoid fractures, which may influence the choice of fixation approach. The dorsal midline approach provides access to most carpal structures and allows assessment of both intrinsic and extrinsic ligament injuries. More recently, several authors have described open reduction combined with arthroscopic assistance for improved visualization and management of carpal injuries [14].

Scaphoid osteosynthesis is most commonly performed using a distal-to-proximal Herbert screw fixation technique. However, in cases associated with scapholunate dissociation, surgical exposure may require a dorsal approach. Several authors advocate a combined dorsal and volar approach to address both bony and ligamentous injuries. Ligament repair is based on direct suturing with anchors, ligament reinsertion when feasible, and temporary Kirschner wire fixation to maintain carpal stability during healing.

The combination of scapholunate and lunotriquetral pinning provides stabilization of the two main sites of post-traumatic carpal instability. This technique offers solid stabilization of the lunate through two fixation points, reducing the risk of secondary rotation around a single pin while preserving radiocarpal joint integrity.

In trans-scaphoid perilunate fracture-dislocations, lunotriquetral pinning contributes to stabilization of the ulnar carpal column. When associated scapholunate dissociation is present, additional scapholunate pinning is required. During wire placement, the pins should be oriented toward the lunate to effectively reduce and maintain correction of scapholunate or lunotriquetral diastasis (Table 2).

**Table 2:** Surgical approaches reported in published series and the present study

Authors	Dorsal Approach	Volar Approach	Combined Approach
Herzberg et al. [4] (13 cases, 1993)	11	0	3
Fikry et al. [15] (39 cases, 1993)	6	13	7
Trumble et al. [11] (22 cases, 2004)	0	0	22
Hildebrand et al. [16] (23 cases, 2000)	0	0	23
Sotereanos et al. [17] (11 cases, 1997)	0	0	11
Inoue et al. [18] (8 cases, 1997)	8	0	0
Martinage et al. [9] (14 cases, 2008)	13	0	1
Laporte et al. [19] (17 cases, 2012)	12	1	0
Israel et al. [20] (65 cases, 2016)	42	7	6
Present series (16 cases, 2017)	12	1	3

When treatment is appropriately performed, healing allows restoration of normal carpal biomechanics, with satisfactory alignment of the carpal bones in both static and dynamic conditions. Associated fractures generally heal in an acceptable position. The duration of immobilization remains an important factor for ligament healing; however, prolonged immobilization may result in joint stiffness. Some authors recommend immobilization for four weeks [3]. No recurrence has been reported in the literature following four weeks of immobilization.

Radiographic condensation of the lunate or proximal pole of the scaphoid is frequently observed during the first one to two years following injury. This finding should not be interpreted as evidence of osteonecrosis. It is usually transient, with progressive restoration of bone density over time, suggesting spontaneous revascularization. Therefore, radiographic condensation alone does not indicate necrosis, and surgical intervention should not be considered in the absence of other clinical indications, even when mild pain persists.

Functional outcomes are generally assessed using clinical scoring systems, including the Cooney score, PRWE, and QuickDASH, Shoulder and Hand questionnaire. Although widely used, these scoring systems have limitations because they rely partly on subjective criteria and do not account for radiological or anatomical changes. A structural abnormality may remain clinically silent initially but become symptomatic later, affecting long-term wrist function.

Functional outcomes reported in the literature are summarized in Table 3. Compared with previous studies, our series had a relatively short mean follow-up duration of 24 months.

**Table 3:** Comparison of functional outcomes reported in the literature and the present series Cooney: modified Mayo Wrist Score; QuickDASH: Quick Disabilities of the Arm, Shoulder and Hand; PRWE: Patient-Rated Wrist Evaluation

Study	No. of Patients	Follow-up (Months)	Flexion-Extension Arc (°, % of Contralateral Side)	Grip Strength (% of Contralateral Side)	Mean Cooney Score (/100)	QuickDASH Score (/55)	PRWE Score (/150)
Trumble et al. [11]	22	49	106° (80%)	77%	75	-	-
Knoll et al. [21]	25	44	113° (83%)	80%	-	-	-
Souer et al. [22]	18	44	73–79°	67–74%	-	-	-
Martinage et al. [9]	14	25	91° (74%)	77%	72	-	-
Lutz et al. [23]	25	60	66%	80%	82	11–14	-
Forli et al. [24]	18	156	95° (75%)	87%	76	-	-
Kremer et al. [25]	39	65,5	77° (63%)	71%	70	23	-
Capo et al. [26]	25	24	82°	59%	-	40	-
Laporte et al. [19]	17	26	101° (77%)	69%	63	24	41
Chou et al. [27]	24	45	144° (90%)	84%	83	-	-
Israel et al. [20]	65	96	96° (69%)	79%	66	21	28
Present Series	16	24	98° (72.5%)	76%	65	23	40

Regarding functional outcomes, our mean Cooney score was 65/100, representing a satisfactory result and remaining consistent with previously reported findings. Studies using the PRWE and QuickDASH questionnaire remain limited. Our mean raw QuickDASH score was 23, which falls within the range reported in the literature (21-40), except for the study by Lutz et al. [23], which reported a lower score of 11. Our mean raw PRWE score was 40, comparable to the score of 41 reported by Laporte et al. [19], but higher than the score of 28 reported by Israel et al. [20].

Reported wrist flexion-extension arcs vary considerably among studies, ranging from 76° to 144°. In our series, the mean flexion-extension arc was 98°. Mean grip strength was 38 kg, corresponding to 76% of the contralateral side. This finding is consistent with previous reports, which describe recovery ranging from 59% to 87% of the contralateral grip strength.

The relatively low rate of advanced degenerative changes observed in our study (four cases, 25%) may be related to the relatively short follow-up duration. The capitolunate articulation, being one of the most congruent joints of the midcarpal region, is frequently involved in perilunate injuries, and articular damage at this level may contribute to long-term degenerative changes. Studies with shorter follow-up periods report lower rates of post-traumatic osteoarthritis, whereas longer follow-up periods demonstrate progressively higher rates. It is also important to note that radiographic deterioration progresses over time. Midcarpal and radiocarpal degenerative changes are frequently observed after these injuries but do not necessarily correlate with clinical symptoms.

Regarding complications, our series included two cases of superficial infection at the pin insertion sites, three cases of Kirschner wire migration, and two cases of paresthesia involving the lateral aspect of the hand. Scaphoid nonunion remains an important complication, particularly in fracture-dislocations managed conservatively. The introduction of surgical fixation has significantly reduced its incidence, although it has not eliminated the risk. Without appropriate treatment, scaphoid nonunion may progress to advanced scaphoid nonunion collapse, characterized by flexion deformity of the scaphoid and dorsal displacement of the proximal scaphoid pole and lunate, ultimately resulting in loss of radioscaphoid congruity and degenerative radiocarpal arthritis.

Proximal pole necrosis of the scaphoid and lunate remains a recognized but relatively uncommon complication. However, no statistically significant factors have been identified to reliably predict its occurrence. Residual scapholunate and lunotriquetral instability following missed diagnosis or inadequate treatment are uncommon, and the coexistence of both instability patterns remains particularly rare.

This study has several limitations. Its retrospective design is inherently subject to selection and information bias. In addition, the absence of a control group precluded direct comparison of different treatment strategies. The relatively small sample size reflects the rarity of perilunate injuries and limits the statistical power of the analysis. Surgical management was individualized according to the injury pattern, resulting in treatment heterogeneity that may have influenced functional outcomes. Furthermore, the mean follow-up duration of 24 months was shorter than that reported in most published series, which may have led to an underestimation of late complications, as post-traumatic osteoarthritis and carpal instability may become apparent only after several years. Finally, the limited number of patients prevented a meaningful statistical correlation between radiographic findings and functional outcomes. Larger prospective studies with longer follow-up periods are required to better define the long-term clinical and radiological prognosis of perilunate injuries.

## Conclusions

Perilunate dislocations and perilunate fracture-dislocations of the carpus are high-energy injuries associated with severe osseous, cartilaginous, and ligamentous damage and may result in long-term complications, including post-traumatic osteoarthritis, carpal instability, persistent pain, and reduced wrist function. In our series, early diagnosis and appropriate surgical management were associated with satisfactory functional outcomes. The main principles of treatment include prompt reduction, stable surgical fixation adapted to the injury pattern, and limited immobilization to achieve ligament healing while minimizing postoperative stiffness. However, the small sample size and relatively short follow-up period limited the ability to establish significant correlations between radiographic findings and clinical outcomes. Further studies with larger cohorts and longer follow-up are required to better define prognostic factors and the long-term impact of radiographic changes on wrist function.

## Appendices

Appendix 1

Data Collection Form

Perilunate Dislocations and Perilunate Fracture-Dislocations of the Carpus

1. Patient Demographics

Patient name:

Admission number:

Age:

Sex:

Address:

Length of hospital stay:

Dominant hand:

Right

Left

Occupation:

Manual worker

Non-manual worker

2. Medical History

Medical history:

Previous surgical history:

3. Injury Characteristics

Mechanism of injury:

Fall

Assault

Road traffic accident

Occupational accident

Sports-related injury

Other

Injury mechanism:

Time from injury to hospital admission:

Affected side:

Right wrist

Left wrist

Bilateral

4. Clinical Assessment

Symptoms:

Functional impairment

Pain

Swelling

Deformity

Other

Physical examination findings:

Associated injuries:

Open injury (Gustilo-Anderson classification):

Type I

Type II

Type IIIA

Type IIIB

Type IIIC

Nerve injury:

Vascular injury:

Associated fractures:

Other associated injuries:

5. Radiological Assessment

Plain Radiographs

Views obtained:

Anteroposterior view

Lateral view

Oblique view

Other

Herzberg Classification

Anteroposterior radiograph:

Pure perilunate dislocation

Perilunate fracture-dislocation

Trans-scaphoid perilunate fracture-dislocation

Distal radius fracture-associated injury

Lateral radiograph:

Dorsal displacement:

Stage I

Stage II

Volar displacement:

Stage I

Stage II

Computed tomography (CT):

Performed

Not performed

6. Treatment

Time from injury to definitive management:

Closed reduction under anesthesia:

Performed

Not performed

Patient positioning:

Type of anesthesia:

General anesthesia

Regional anesthesia

Other

Surgical approach:

Percutaneous

Volar approach

Dorsal approach

Combined volar and dorsal approach

Fixation methods:

Kirschner wires (K-wires)

Screws

Other fixation methods

Bone grafting:

Yes

No

Postoperative immobilization:

Type:

Duration:

Postoperative management:

Antibiotic prophylaxis:

Rehabilitation:

Delay before initiation:

Self-directed rehabilitation

Supervised rehabilitation

7. Follow-Up and Outcomes

Hardware Removal

Time to hardware removal:

Complications

Minor complications:

Complex regional pain syndrome

Residual pain

Wrist stiffness

Major complications:

Carpal tunnel syndrome

Pin-site infection

Kirschner wire migration

Scaphoid nonunion

Osteonecrosis:

Scaphoid

Lunate

Post-traumatic osteoarthritis

Follow-Up Duration

Follow-up period (months):

Functional Outcomes

Wrist mobility:

Mean flexion-extension arc:

Mean radial/ulnar deviation:

Mean pronation-supination:

Grip strength:

Mean grip strength:

Comparison with contralateral side:

Weight-lifting capacity:

Functional scores:

Modified Mayo Wrist Score (Cooney score):

Excellent

Good

Fair

Poor

Patient-Rated Wrist Evaluation (PRWE):

Quick Disabilities of the Arm, Shoulder and Hand questionnaire (QuickDASH):

Return to Work

Time to return to work:

Return to previous occupation:

Occupational reassignment:

Loss of employment:

Pain Assessment

Pain at rest:

Pain during activity:

Visual Analog Scale (VAS) score:

Radiological Outcomes

Scapholunate diastasis:

Carpal height ratio:

Ulnar translation of the carpus:

Post-traumatic osteoarthritis:

Scapholunate advanced collapse (SLAC)

Scaphoid nonunion advanced collapse (SNAC)

Bone density changes:

Lunate

Scaphoid

Osteonecrosis:

Scaphoid

Lunate
